# Characterization of the Maternally Derived Antibody Immunity against Rhdv-2 after Administration in Breeding Does of an Inactivated Vaccine

**DOI:** 10.3390/vaccines8030484

**Published:** 2020-08-28

**Authors:** Massimiliano Baratelli, Joan Molist-Badiola, Alba Puigredon-Fontanet, Mariam Pascual, Oriol Boix, Francesc Xavier Mora-Igual, Michelle Woodward, Antonio Lavazza, Lorenzo Capucci

**Affiliations:** 1 HIPRA, 17170 Amer, Spain; joan.molist@hipra.com (J.M.-B.); alba.puigredon@hipra.com (A.P.-F.); oriol.boix@hipra.com (O.B.); michelle.woodward@hipra.com (M.W.); 2 Institute of Agrifood Research and Technology (IRTA), 08140 Barcelona, Spain; mariam.pascual@irta.cat; 3 Asvet Veterinaris, 08410 Barcelona, Spain; asvetveterinaris@gmail.com; 4 Istituto Zooprofilattico Sperimentale della Lombardia e dell’Emilia-Romagna (IZSLER), 25124 Brescia, Italy; antonio.lavazza@izsler.it (A.L.); lorenzo.capucci@izsler.it (L.C.)

**Keywords:** RHDV-2, inactivated vaccines, maternal derived immunity, rabbits

## Abstract

Inactivated strain-specific vaccines have been successfully used to control rabbit haemorrhagic disease (RHD) caused by RHDV-2 in the rabbit industry. It is unknown whether and how vaccination of breeding does contributed to protect the population of young susceptible rabbit kits. The present study investigates whether the immunity against RHDV-2 produced by vaccination of breeding does is transmitted to their progeny and its dynamic once inherited by kits. For this purpose, New Zealand female rabbits of 8–9 weeks of age were allocated into 2 groups of 40 subjects each and bred during 6 reproductive cycles. The first experimental group was vaccinated with a commercially available inactivated vaccine against RHDV-2 whereas the second group was inoculated with PBS. Moreover, the present study was also meant to identify the mechanisms of transmission of that maternal immunity. For this reason, rabbit kits of vaccinated and non-vaccinated breeding does were cross-fostered before milk uptake. The RHDV-2 antibody response was monitored in the blood serum of breeding does and of their kits by competition ELISA (cELISA) and solid-phase ELISA (spELISA). Since it has been clearly demonstrated that cELISA positive rabbits are protected from RHD, we avoided the resorting of the challenge of the kits with RHDV-2. Results showed that RHDV-2 antibodies were inherited by kits up to one year from vaccination of breeding does. Once inherited, the maternally derived antibody response against RHDV-2 lasted at least until 28 days of life. Finally, the study also elucidated that the major contribution to the maternal derived immunity against RHDV-2 in kits was provided during gestation and probably transmitted through transplacental mechanisms although lactation provided a little contribution to it. The present study contributed to elucidate the characteristics of the maternal antibody immunity produced by vaccination and its mechanisms of transmission.

## 1. Introduction

Rabbit haemorrhagic disease (RHD) is considered a serious concern for rabbit industry and a major threat to wild rabbit fauna. Since its first detection in 1984 and up to 2010, the RHDV (genotype GI.1) and its variant RHDVa (genotype GI.1a) had been the unique known aetiological agents responsible of the disease. RHDV causes an acute hepatitis with a case fatality ratio higher than 80% in adult rabbits while the infection is asymptomatic in rabbits less than 6–7 weeks old [1]. The European rabbit (Oryctolagus cuniculus) is the only host of RHDV. The emergence of a new Lagovirus, RHDV type 2 (RHDV-2, genotype GI.2) in 2010 dramatically changed the epidemiology of this disease [2,3]. This was due to the unique characteristics of RHDV-2: (a) it is a distinct serotype from RHDV, (b) it causes the disease also in young rabbits, (c) its host spectrum is much broader than the RHDV spectrum, including more species of hares and other lagomorphs [4,5]. Collectively, these characteristics allowed RHDV-2 to replace RHDV/RHDVa causing devastating outbreaks and rapidly becoming endemic in Europe, Asia, Africa and Australia. Recently, its broader host spectrum made RHDV-2 to became endemic also in the wild fauna of north and central America [6], while RHDV/RHDVa, since 2000, have caused just rare outbreaks, probably due to reintroductions from other continents.

Inactivated vaccines against RHDV and more recently for RHDV-2, have been developed and successfully used to control the disease in the rabbit industry. These vaccines work efficiently because they promptly and highly stimulate the humoral immunity (i.e., antibodies response) that is the main defensive mechanism against RHD [7,8,9,10,11,12]. Despite this, almost thirty years later, some questions about the immunization properties of those inactivated vaccines are still open. In fact, these vaccines were licensed to protect adult rabbits (weaned) by means of active immunization, so they were not designed to protect younger animals. Therefore, the overall immunisation capacity and the mechanisms by which they contribute to protect the population of rabbit kits are still unclear. 

In rabbit wild populations, maternal derived immunity is considered an important factor contributing to protect against RHD or delaying the infection so reducing the severity of outbreaks [13]. In mammals, maternal derived antibodies (MDA) are a type of passive immunity transmitted from mothers to offspring during the gestation and/or lactation and which generally aid to protect during their early life. Rabbits have a haemochorial placentation and thus it is known that maternal antibodies are transmitted from the mother to the offspring through placenta [14]. Evidences of transmission during lactation have been provided but no much is known about the related mechanisms [14,15]. Rabbits have only one Cγ gene which means they have only one IgG subclass [16], as consequence, no restriction of the subclass of the IgG is expected during maternal transfer. 

Differently from RHD caused by RHDV which induces clinical disease and mortality only in animals older than two months of age, in outbreaks caused by RHDV-2 even very young animals (10–15 days of age) can be infected and die. Therefore, the maternal immunity generated by vaccination might be implicated in protecting rabbit kits against the disease produced by RHDV-2. 

The aim of this study was to test whether an inactivated vaccine indicated for active immunization of adult rabbits can produce a passive antibody immunity in blood against RHDV-2 in rabbit kits. Beside this, it was also meant to elucidate the mechanisms of the RHDV-2 antibody immunity transmission and to evaluate its dynamic once inherited by kits. Finally, this study was meant to evaluate whether revaccination of breeding does is needed to increase passive immunity of rabbit kits during their life-span.

## 2. Materials and Methods 

### 2.1. Animals and Facilities

Female New Zealand White rabbits of 8–9 weeks of life (wol) were purchased from a minimal disease grade animal supplier (Granja San Bernardo, Navarra, Spain). The supplier herd was free from mayor rabbit diseases including RHD. Rabbits were housed in an experimental facility (IRTA, Torre Marimon, Barcelona, Spain) and reared in individual cages. Feed and water were administered ad libitum. Rabbits were vaccinated 4 weeks after the arrival at the facilities and then revaccinated each 4 months with MIXOHIPRA^®^-FSA (HIPRA, Amer, Spain). Myxomatosis is an immunosuppressing disease of rabbit, which is endemic in Spain; rabbits were vaccinated to avoid eventual interference of the disease with the study. The animal procedures were approved by animal care and use committee of the Institut de Recerca i Tecnologia Agroalimentàries (IRTA) (reference FUE-2018–00760718 and ID 557G1BW29). Following that approval and with the aim to meet general requests to avoid, as much as possible, the use of animal in scientific experiments, we did not perform virulent challenge in kits. This was planned even considering that previous studies demonstrated that rabbits with positive antibodies titres in cELISA resulted protected from RHD caused by the homologous RHDV [7,8,9,10,11,12].

### 2.2. Experimental Design

ERAVAC^®^ (HIPRA, Amer, Spain) is a vaccine formulated with an inactivated RHDV-2 virus isolated from an outbreak in Spain (strain V-1037) and a mineral oil adjuvant (water-in-oil-in-water emulsion). RHDV-2 is only able to replicate in live infected rabbits and thus the virus is normally recovered from their livers and spleens when they have succumbed to the infection or are euthanized. Direct measurement of the quantity of the inactivated virus in the vaccine is not easily achieved and other components may interfere with currently available assay methods. Therefore, the potency of the vaccine per dose is expressed as a minimum 70% of vaccinated rabbits giving a cELISA (competitive enzyme-linked immunosorbent assay) serological titre ≥ 40 ELISA Units. Each batch of the vaccine is therefore released to produce at least the cited protective immune response in rabbits. Healthy rabbits were selected and randomly allocated into 2 groups of 40 subjects each. Group A was vaccinated with ERAVAC^®^ (HIPRA) whereas group B was inoculated with sterile Phosphate-Buffered Saline (PBS). Vaccination was performed after one week of acclimation (at 9–10 wol) by subcutaneous administration of 0.5 mL of the products. The immunization status of all rabbits was checked 25 days later; for this purpose, blood was collected from the central auricular artery and the presence of antibodies against RHDV-2 was determined on the separated sera. The breeding program started at 17–18 wol (56 days post vaccination) and continued up to six reproduction cycles (almost 351 days post vaccination, see Table S1). Oestrus synchronization and superovulation were induced with Pregnant Mare Serum Gonadotrophin (PMSG), 35IU (International Unit), 48 h before the insemination. The insemination was performed artificially with semen from mixed males (IRTA). The interpartum period was between 49 and 56 days. After starting the breeding program, the experiment was split into three different parts. 

### 2.3. Experimental Design Part 1

The duration of the immune response in breeding does was monitored up to 351 days post vaccination (dpv). This time frame permitted to study the maternal immunity transmission during six reproductive cycles. Ten breeding does were selected among those having continuous successful parturitions at the second, third, fourth and sixth reproductive cycle. Blood samples were collected from the central auricular artery of the animals at 2 days after the parturition. The presence of maternal derived antibody response was simultaneously evaluated in kits. For this purpose, kits of 2 days of life (dol) from the 10 above selected mothers were bled intracardiacally; animals were previously administered with anaesthetics drugs (Xylazine 0.2 mg/kg and tiletamine-zolazepam 0.2 mg/kg) whereas after the procedure they were humanely euthanized with an overdose of sodium pentobarbital (200 mg/kg, intracardiac). 

### 2.4. Experimental Design Part 2

The duration of the maternal derived immunity was monitored up to 58 dol which was considered similar to a common life cycle length of meat producing rabbits. Forty rabbit kits per group born from groups A and B at the second reproduction cycle were randomly selected, weaned at 30–35 dol and grown up to 60 dol. Maternally derived immune response was monitored during this period. Blood samples were collected periodically (2, 15, 28, 40, 51, 58 dol) either by intracardiac puncture or from the central auricular artery of 15 kits per group.

### 2.5. Experimental Design Part 3

The mechanisms of maternal immune transmission were determined by cross-fostering rabbit kits of 6 breeding does of groups A with those of group B and then monitoring their RHDV-2 antibody response. Birth was induced in breeding does at the third reproduction cycle by using 5 UI of oxytocin HORMONIPRA (HIPRA). The procedure was carried on just after birth but before the start of the maternal milk intake. The group of rabbit kits that was moved from group A to B was called AB. In contrast, the opposite group was called BA. The cross-fostering was also performed between kits born from breeder does of the same group to evaluate the influence of possible confounding bias associated to the implemented procedures; for this purpose, the same process was performed among 4 mothers of group A and also among 4 mothers of group B. The derived groups of rabbit kits were respectively called AA and BB. Maternal derived immunity was monitored in the rabbit kits at 2, 9, 19 and 29 dol; for this purpose, between 4–12 randomly selected kits per group were bled either by intracardiac punctuation or from the central auricular artery.

### 2.6. Detection and Quantification of Antibodies Against Rhdv-2

Rabbit sera were tested using two different ELISAs: (a) a competition ELISA (cELISA RHDV-2) to test the presence of Ig (IgG, IgM and IgA) anti RHDV-2; (b) a solid phase ELISA (spELISA) to test the presence of IgG anti RHDV-2. In case of positive sera, the semi-quantitative level of antibodies was established in both tests. In both ELISAs sera and reagents were diluted in PBS with 1% yeast extract and 0.05% Tween 20 pH 7.4 and OPD (o-Phenylenediamine dihydrochloride) was used as a soluble substrate for the detection of peroxidase activity. cELISA was already described [5,11]. Briefly, rabbit IgG purified from an anti RHDV2 high titre serum, were adsorbed overnight at 4 °C to the solid phase at 2 μg/mL in carbonate buffer pH 9.6. In the second step, sera were diluted starting from the 1/10 dilution in four folds dilution in presence of a fix amount of antigen (RHDV-2) used as limiting reagent. Finally, the monoclonal antibody (MAb) anti RHDV 4H12 HRP conjugated where used to detect the RHDV-2 bound to the well. A serum sample was considered negative if its OD value at the 1/10 dilution was higher than 85% of the optical density (OD) value at the same dilution of the negative control serum. Serum was considered doubtful (inconclusive result) if its OD value at the 1/10 dilution was equal to or higher than 75% of the OD value at the same dilution of the negative control serum. A serum sample was considered positive if its OD value at the 1/10 dilution was lower than 75% of the OD value at the same dilution of the negative control serum. The titre of a positive serum sample corresponded to the dilution causing a 40–60% reduction of the OD value of the negative control serum.

In spELISA semi purified RHDV-2 was adsorbed at a predefined dilution (established by ELISA titration) directly to the solid phase in PBS pH 7.4, incubating the plate overnight at 4 °C. In the second step sera were diluted starting from 1/40 in four folds dilution and incubated for 1 h at 37 °C. Finally, the MAb 4H9 anti IgG rabbit HRP conjugated was used in the last step. In each run 3 distinct negative control sera were included, and the mean OD obtained for each dilution was subtracted to those of the tested sera (background subtraction). A serum was considered positive if the OD at the dilution 1/40 was higher than 0.15 OD. The titre of a positive serum sample corresponded to the dilution of the serum with an OD value in the rage 0.15–0.25 OD. 

### 2.7. Statistical Analysis

Data collation, initial analysis and graphs generation were performed using Excel (Microsoft). For analysis purposes, doubtful results were considered negative and negative results were transformed as 1 reciprocal antibody titre. The reciprocal antibody titres were then transformed as logarithm on a 2 base. Statistic comparisons between groups of the Log_2_ transformed antibody titres were performed by Kruskal-Wallis or Mann-Whitney U tests because the data were not normally distributed. Statistic comparisons between groups of the number of seropositive rabbits were analysed by Chi-square or Fisher’s Exact Probability tests whether the lowest expected frequency was respectively superior to 5 or lower. The interaction effect between the Log_2_ transformed antibody titres and the days post vaccination in group A was estimated by the one-way ANOVA for repeated measures. All tests were implemented in IBM SPSS Statistics (IBM Corp., Armonk, NY, USA) and performed with a significance level of 5%. The simple linear regression and estimation of coefficients was performed by IBM SPSS Statistics. The half-life was calculated as the time needed to decrease 1 Log_2_ of antibody titres.

## 3. Results

### 3.1. Passive Immunization of Rabbit Kits by Means of Active Immunization of Breeder Does

**Figure 1:**
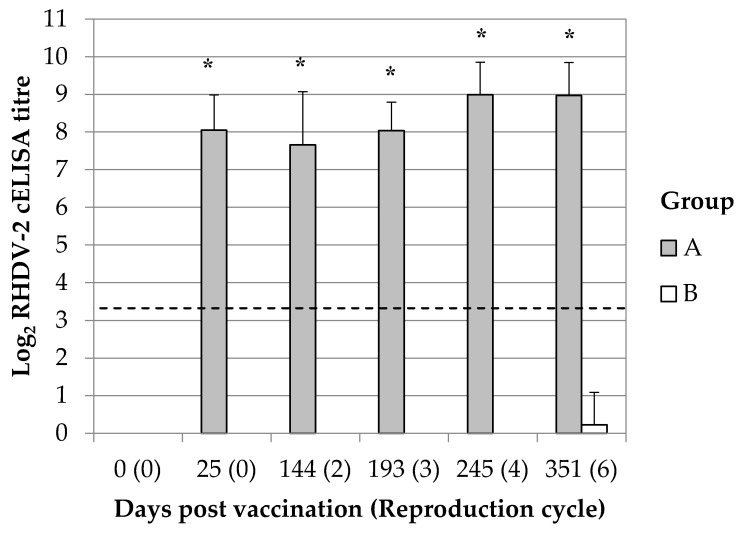
RHDV-2 antibody response in breeding does. Results are represented as average and standard deviation. Breeding does were vaccinated either with ERAVAC^®^ (Group A) or inoculated with PBS (Group B). The slashed line indicates the cut-off of the assay (3.32 Log_2_ cELISA titre). Asterisks (*) indicate statistically significant differences between groups (Mann-Whitney U test, *p* < 0.05).

Vaccination produced in breeding does an antibody immune response against RHDV-2 which was detectable by the cELISA at 25 dpv, the first sampled time point in the study protocol, and which persisted up to 351 dpv (Figure 1). Group A showed an average of 8.05 ± 0.93 Log_2_ cELISA titre at 25 dpv; then, the sampled breeding does showed average titre ranging between 7.66 and 8.99 Log_2_ cELISA. The antibody response was not maintained constant during the entire study (ANOVA, F(4, 77) = 5.324; *p* = 0.001) and unexpectedly it slightly increased after 200 dpv. Group B animals were maintained negative until the fourth reproduction cycle; later on, few breeding does showed a very low antibody response against RHDV-2 which was just above the threshold of the assay (3.32 Log_2_ cELISA titre). The observed results were considered not specific, probably due to the aging of animals. The RHDV-2 antibody response of breeding does of group A and B was statistically significantly different since 25 dpv and up to the end of the study (Mann-Whitney U test; *p* < 0.05).

**Figure 2:**
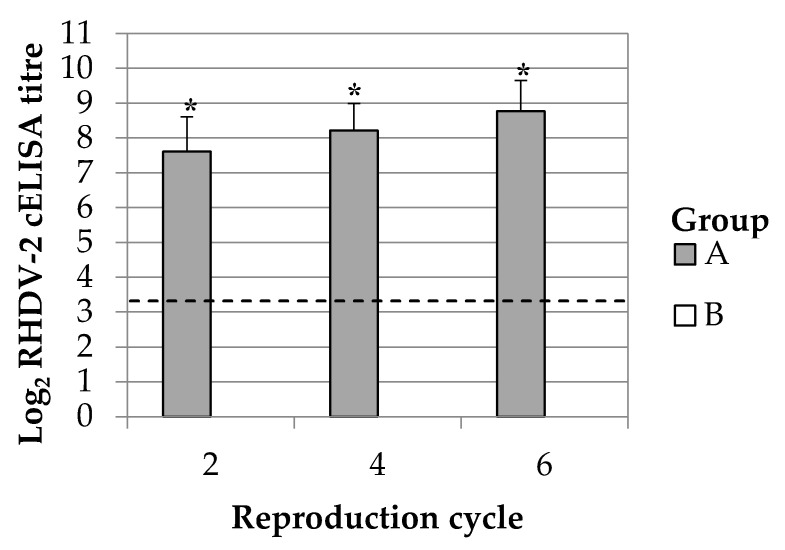
RHDV-2 antibody immunity in rabbit kits of 2 days of life. Results are represented as average and standard deviation. Breeding does were vaccinated either with ERAVAC^®^ (Group A) or inoculated with PBS (Group B). The slashed line indicates the cut-off of the assay (3.32 Log_2_ cELISA titre). Asterisks (*) indicate statistically significant differences between groups (Mann-Whitney U test, *p* < 0.05).

Vaccination of breeding does produced a passive antibody response in their kits. All rabbit kits produced by group A showed presence of antibodies against RHDV-2 after every inspected reproduction cycle whereas no positive rabbit kit was observed in group B (Figure 2). The average titres at 2 days of life were ranging between 7.61 and 8.77 Log_2_ cELISA. Paired comparison between breeding does and their rabbit kits cELISA titres showed that on average their base 2 logarithmic difference was 0.93 ± 0.11.

### 3.2. Duration of the Maternally Derived Antibody Immunity Produced by Vaccination in Rabbit Kits

**Figure 3:**
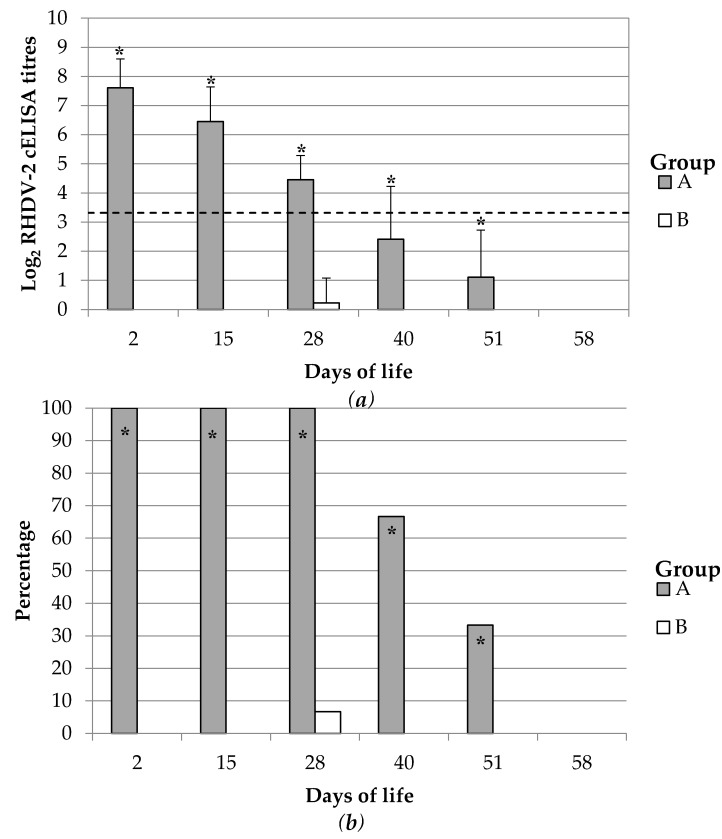
Duration of the maternal antibody immunity against RHDV-2 in rabbit kittens. Results are represented as (**a**) average and standard deviation of Log2 cELISA titres and (**b**) as percentage of positive rabbits. Rabbit kittens were born from breeding does vaccinated with either ERAVAC^®^ (Group A) or inoculated with PBS (Group B). The slashed line indicates the cut-off of the assay (3.32 Log_2_ cELISA titre). Asterisks (*) indicate statistically significant differences between groups ((**a**) Mann-Whitney U test, *p* < 0.05; (**b**) Chi-square or Fisher test, *p* < 0.05).

The duration of the antibody immunity against RHDV-2 produced by vaccination of breeding does in rabbit kits was monitored during an entire productive life cycle. This lasted at least 28 days from birth but did not reach 58 days (Figure 3). The rabbit kits born from group A showed an initial average cELISA titre of 7.61 ± 0.99 Log_2_ which then decreased in a time dependent manner. The average antibody titre was maintained above the cut-off of the cELISA up to 28 dol; subsequently, it further decreased, and thus negative rabbit kits were firstly observed at 40 dol. None of the inspected rabbit kits was positive at 58 dol. Almost all rabbits born from non-vaccinated breeder does were maintained negative during the entire study. One kitten showed RHDV-2 titres close to the cut-off (3.32 Log_2_ cELISA titres) at 28 dol; however, the same rabbit was negative further on during the study and the other kits of the same litter were negative. 

The spELISA was used to determine the duration of the maternal derived antibody immunity against RHDV-2 in rabbit kits considering its higher sensitivity compared to the cELISA (Figure S1). Results showed that the average Log_2_ spELISA titres were still above the detection limit of the assay at 40 dol but first negative rabbits were also observed by that time. Therefore, results confirmed that the maternal derived immunity against RHDV-2 in rabbit kits had a duration of at least 28 days. 

The antibody immunity in rabbit kits blood decreased in a time dependent manner, as observed above. The half-life and thus the pace at which the RHDV-2 cELISA and spELISA antibodies titres wane was calculated from the data obtained up to 28 dol, when the average values were still above the cut-off. As first step a simple linear regression was calculated (Figure S2 and Table S2) to predict Log_2_ cELISA and Log_2_ spELISA titres based on the days of life; then, the half-life was calculated using the linear regression line coefficient as the time needed to have 1 Log_2_ less (50% of the reciprocal) of the initial antibody titre. The RHDV-2 antibodies in rabbit kits showed a half-life of 8.2 (6.62–10.87 95%CI) and was 5.49 (4.42–7.19 95%CI) days when determined by the cELISA and the spELISA respectively.

### 3.3. Mechanisms of Transmission of the Maternally Derived Antibody Immunity

The mechanisms of maternal antibody transmission and their relative relevance were determined by cross-fostering rabbit kits between breeding does vaccinated with either ERAVAC^®^ or inoculated with PBS. Moreover, kits born from breeding does of the same group were also cross-fostered to check the confounding effects of this procedure. 

**Figure 4:**
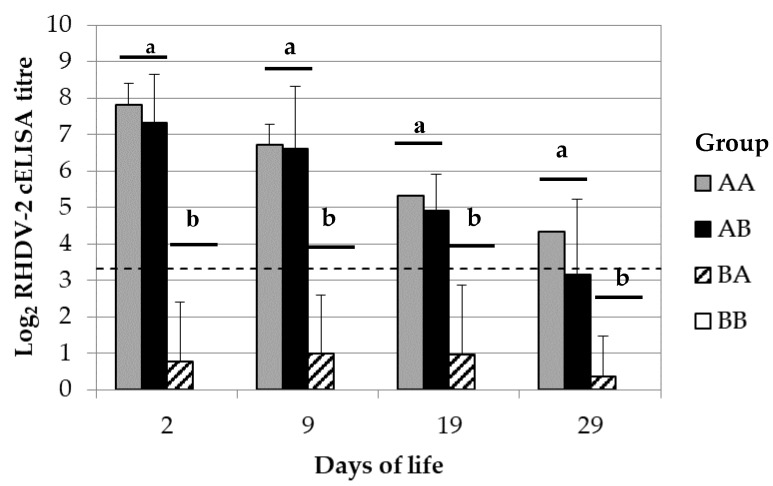
Mechanism of RHDV-2 antibody transmission from breeding does to rabbit kittens. Results are represented as average and standard deviation of Log_2_ cELISA titres. The rabbit kittens were cross fostered between ERAVAC^®^ (AA), PBS vaccinated breeding does (BB) or between them (AB, BA). The slashed line indicates the cut-off of the assay (3.32 Log_2_ cELISA titre). Different letters indicate statistically significant difference between groups (Kruskal Wallis test *p* < 0.05).

The rabbit kits taken from ERAVAC^®^ vaccinated and moved to PBS inoculated breeding does (group AB) showed similar titres of RHDV-2 antibodies in sera compared to group AA (Figure 4). On the other hand, the rabbit kits moved from PBS to ERAVAC^®^ vaccinated breeding does (BA) showed lower titres of RHDV-2 antibodies in sera compared to AA and AB but similar to group BB (Figure 4). Most rabbit kits in group BA were negative; nevertheless, a few of them (1–3 kits) were found positive sporadically alongside the study and with titres close to the cut-off (3.32–4.32 Log_2_ cELISA). 

The results obtained from the control groups AA and BB were consistent with those obtained from the previous parts of this study. The group AA was constituted by rabbit kits born from breeding does vaccinated with ERAVAC^®^ and cross-fostered between them. Their titres of RHDV-2 antibodies in blood at 2 days of life (7.82 ± 0.58 Log_2_ cELISA) were similar to the rabbit kits born from group A at the second reproduction cycle (Mann-Whitney U test, *p* > 0.05); therefore, the cross-fostering procedure had no detectable confounding effect on the maternal transmission of the antibodies. On the other hand, the rabbit kits born and cross-fostered between PBS inoculated breeding does did not show detectable RHDV-2 antibodies during the entire study (Figure 4). 

Again, the spELISA was used to determine the duration of the maternal derived antibody immunity against RHDV-2 in rabbit kits with a higher sensitivity compared to the cELISA (Figure S3). The results of groups AA and AB were in line with the above finding; instead, group BA showed average titre above the cut-off of the assay. Furthermore, most of the rabbit kits were positive throughout the study (between 60% and 100%) and those showed titres ranging between 5.32 and 9.32 Log_2_ spELISA.

## 4. Discussion

It is unclear whether inactivated vaccines licensed to protect against RHD can also produce a passive immunity in rabbit kits. In the present study, vaccination with an inactivated vaccine produced, as expected, a long-lasting antibody response against RHDV-2 in breeding does which was maintained up to a year after its administration. The rabbit kits born from these vaccinated does showed the presence of a RHDV-2 specific antibody response which waned over time. In contrast, PBS inoculated rabbit does and their kits were maintained negative suggesting that the detected immunity was specific of the vaccine and not produced by an infection with RHDV-2. Rabbits of less than 2–4 weeks of life are generally unable to mount a normal specific immune response to antigens [17]. Based on this and the above results it can be suggested that the detected immunity in rabbit kits of this study was not active but instead it was derived from their mothers. Therefore, the used inactivated vaccine demonstrated producing a passive antibody immunity in rabbit kits by means of active immunization of the breeding does. Previous authors demonstrated that the maternally derived immunity produced by infection contributes to protect against RHD. Rabbit kittens with RHDV cELISA titres above 1/10 were showed to be protected against challenge infection [12] (C.L. personal communication). This is supported from further studies that showed that rabbits positive in cELISA, even at low titres up to 1/10, resulted protected from RHD when challenged with the homologous RHDV [7,8,9,10,11,12]. However, this parameter did not fully correlate with protection as also few rabbit kittens with values falling below the cELISA cut-off were protected [12]. Therefore, in the absence of a challenge experiment of kits with RHDV2, we can just conclude that kits are protected by RHD at least when they show titre of 1/10 in cELISA. Moreover, in absence of challenge experiments, we cannot state if kits, in addition to being protected from disease, are also protected from infection. In fact, this could only be ascertained by post challenge serological analysis (i.e., is there seroconversion or not with the appearance of IgM?). However, it is useful to remember that in paper of Robinson et al. [12] it was proved that kits with ELISA titres ≥ 1/60 were protected also from infection.

In rabbits, passive immunization is not a common procedure and there is no standard program. The maternal IgG immunity transfer occurs mainly during gestation and primarily across the visceral yolk sac of rabbits. Furthermore, most of IgG antibodies are transferred during the second half of gestation (15 days of gestation) [18]. Based on this information and under experimental settings, vaccines have been generally administered to rabbits just before or during gestation to produce a passive immunity [15,19,20]. Immunizing at each gestation is not feasible in rabbit industry; breeding does undergo several reproduction cycles which would imply revaccinating 6–7 times in a single year. In the present study, the administration of a single dose of the vaccine to breeding does demonstrated to be enough to produce a passive antibody immunity against RHDV-2 in rabbit kits up to the sixth reproduction cycle and thus almost a year later. This finding suggests that revaccinating breeding does during each gestation is not needed to produce a passive antibody immunity in rabbit kits and thus more feasible vaccination plans can be designed for field uses. 

The maternal derived antibody immunity is considered to serve to protect kitten rabbits until they can mount a proper immune system and thus until they can produce their own active immune response after vaccination or infection. Therefore, is fundamental that the immunity last until the end of this early period of susceptibility. The active immunization of breeding does with an inactivated vaccine against RHDV-2 showed here to produce a specific antibody response in rabbit kits which lasted at least 28 days and thus persisted until the end of the susceptibility window. As far as we know, this study is the first report describing the complete dynamic of an RHDV-2 specific maternally derived antibody immunity. 

Previous observational studies of wild rabbit populations naturally infected by RHDV (GI.1) estimated that the maternal antibody immunity might last at least 30–37 days of life [11,13] and that most of rabbits lost it before the 56–70 days of life [13]. This length approximates to what observed in the present study suggesting that the maternal antibody immunity against RHD might have a similar duration regardless the genotype of the causing virus or whether it is due to vaccination or infection. In contrast, another study showed that a longer duration of the maternal antibody immunity against RHDV might be achieved in wild populations of rabbits when the natural infection produces higher titres of antibodies in the does [12]. Moreover, the same study found a positive correlation between the levels of antibodies in does and the survival of their rabbit kittens. Similar results were obtained by testing sera of kittens born from convalescent does surviving a natural RHD outbreak and showing very high titres (> 1:2560) i.e., the titres found in kittens were proportionally higher and lasted for a longer period (C.L. and A.L. personal observations).

Despite this, previous other authors estimated that the duration of maternally antibody derived immunity was about 20–22 days of life [15,19] and that the first 10 days of life are characterized by a drastic decrease of its levels [15]. However, in these experiments the passive antibody immunity was produced by vaccination with an RHD unrelated experimental antigens; furthermore, the overall and not the antigen specific IgG immunity was explored in one of those studies. These findings are not consistent with what is here and previously described about RHD. Factors like the initial level of the maternally derived antibody immunity, its half-life, the antigen specificity or the assay used might be important causes of variation. 

The transfer of maternal immunity is an efficient process and, in fact, from the 24th day of gestation the antibody levels in neonatal rabbits approximates those of their mothers [18]. Previous studies showed that new-born rabbit kits passively immunized had levels of antigen specific maternal IgG antibodies similar or slightly varying above or below the levels of their breeding does with differences reaching almost half of the concentration [15,19]. In the present study, the average difference between the breeding does and their rabbit kits was less than a base two logarithm and thus supported the previous studies; therefore, it can be suggested that generally the maternally derived antibody immunity is consistently transferred to rabbit kits. Based on this, maintaining a proper immunization status in breeding does is crucial in order to steadily produce a passive immunity in rabbit kits. Moreover, periodical determination of the blood antibody immunity in breeding does might be a good approximation to estimate the status of the maternal antibody immunity of the future progenies.

The duration of the maternal derived immunity depends on its initial levels but also on the pace at which it waned over time. In the present study, maternally derived immunity against RHDV-2 showed a half-life of approximately 8 or 5 days depending on the detection assay used. The data used to estimate this value were not collected through a longitudinal monitoring of each animal because the bleeding practice required to sacrifice those younger than 30 days of life. Therefore, it was not possible to provide a precise estimation of this parameter. As far as we know, the half-life of maternally derived IgG against RHDV-2 or any other antigens has not been estimated before. Nevertheless, this might be roughly estimated as 7–9 days from data of previous published study [15,19]. More precise studies have been previously conducted to estimate the half-life of IgG in blood of rabbits although these were obtained thorough transfusion and thus not through maternal transference mechanisms; in these studies, human transfused and rabbit IgG showed a half-life of approximatively 5 days in rabbits blood [21,22,23]. These findings suggest a lack of consistency of the IgG half-life in rabbit which might depend on the assay used or on the antigen specificity of the IgG but mostly important also on the immunization model. Nevertheless, the half-life calculated in this study is still important; this might allow generating models to approximately predict the duration of the maternal derived antibody immunity or its level at a specific age by just knowing the initial levels in the new-born rabbit kits or, in case of missing, by even using the breeding does blood levels. 

It is widely accepted that in rabbit the maternally derived antibodies are transmitted to the blood of offspring during gestation and through the placenta [14]; nevertheless, the lactation can also contribute to this process [15]. In the present study, the rabbit kits passively immunised with the vaccine and which lactation was sustained by non-immunised breeding does, showed a maternally derived antibody immunity against RHDV-2 similar to those passively immunised and reared with their own breeding does. In other words, the restriction of the eventual maternally derived IgG antibody immunity supplied during lactation did not significantly changed the levels in rabbit kitten blood. These results suggest that most of the maternal derived antibody immunity against RHDV-2 was transferred during gestation, being in line with the above concept. 

The kitten rabbits non passively immunised but which lactation was sustained by immunised breeding does showed that their average levels of maternally derived antibody immunity against RHDV-2 were not significantly different from the negative control; despite this, some of them were found positive against RHDV-2 and their average levels were increasing over time, although not significatively. Under similar settings, previous authors observed that the maternally derived antibody immunity provided only through lactation increased over time until 16 days of life, with an abrupt drop thereafter [15]. Moreover, by making a rough estimation from the data provided by [15] (Compare Figure 2 and Figure 4), it could be asserted that the maximum amount of antibodies levels reached toward the end of lactation was less than ten times the usual levels. These results suggested a minimal amount of maternally derived immunity against RHDV-2 might have been supplied throughout the lactation period. Nevertheless, this was barely detectable with the available assays. Unfortunately, the antibodies against RHDV-2 were not quantified in milk in this study; therefore, it is not clear whether the finding was related to a low absorption or a low amount of the antibodies in the intestine.

The dynamic observed in this study and by previous authors suggests that the process of IgG uptake from milk in rabbits might be different to those of species like swine and ruminants. In these latter, most of IgG are uptake by off-springs through colostrum during the first 24–48 h of lactation and then their levels waned over time. Instead, rabbits show a dynamic similar to rats in which the uptake of IgG increases over time, reaches the maximum levels at 14 days of life and by the time of weaning it ceases [24]. In accordance with this, the receptors in charge of the IgG uptake (Fc receptor) are highly expressed in the duodenum of rats since the birth and up to the weaning period (approximately 19 days of life) [24]. The knowledge about FC receptor distribution and persistence in the rabbit intestine is missing and it deserves investigation to clarify the transmission mechanisms of IgG during the lactation of rabbit kits. 

Lacteal secretions have an important role in the protection of new-borns during their early life not only because of the transfer of the IgG to blood, in certain species, but also due to the local protection provided at the gastrointestinal tract level [25]. Mucosal IgA are also provided at the same location [25] and might have a role in protection. Although it is important to confirm the existence of the transmission of the maternally immunity to the rabbit kitten blood by lactation, it is also important to clarify its contribution to the protection against RHD considering also the rest of the maternal immunity provided locally at the mucosa of the gastrointestinal tract, one of the site of entry of the virus. 

## 5. Conclusions

The present study demonstrated that an inactivated vaccine designed for active immunization of adult rabbits can produce a passive antibody immunity against RHDV-2 in rabbit kits without implementing a tailor-made administration program. Moreover, the produced immunity is likely to cover the early life of rabbits at least until they turn suitable to be vaccinated (older than 30 days of life). Based on previous studies which showed that rabbits positive in cELISA resulted protected from RHD when challenged with the homologous RHDV, we could conclude that the vaccine used here produce a protective maternal antibody immunity against RHDV-2. However, future challenge infection experiments could be helpful to enforce such previously observed correlation and further confirm it protective extents with mayor sensitivity.

Finally, the present study provides also novel insight into the mechanisms of maternal immunity transmission in rabbit and its dynamic once inherited. It highlights the importance of the transplacental mechanisms and supports the existence of transmission throughout the entire lactation. 

## Supplementary Materials

The following are available online at https://www.mdpi.com/2076-393X/8/3/484/s1, Table S1: Scheme of the experimental design, Figure S1: Duration of the maternal antibody immunity against RHDV-2 in rabbit kittens (spELISA), Figure S2: Dynamic of RHDV-2 antibodies in kittens; simple linear regression, Table S2. Simple linear regression coefficients. Figure S3: Mechanism of RHDV-2 antibody transmission from breeding does to rabbit kittens (spELISA).
