# Supplementary material for: Characterization of the Maternally Derived Antibody Immunity against Rhdv-2 after Administration in Breeding Does of an Inactivated Vaccine
Source: Vaccines (Basel). 2020 Aug 28;8(3):484. doi: 10.3390/vaccines8030484 (PMC7564433; doi:10.3390/vaccines8030484)
Supplement: Supplementary file 1 [file vaccines-08-00484-s001.pdf]

Table 1. Scheme of the experimental design.

wol	9–10	12–13	17–18	21–22	29–30	36–37	43–44	51–52	59–60
dpv	0	25	56	86	142	191	242	296	349
	Vaccination	Bleeding	1st AI [1]	1st		2nd 3rd	4th	5th	6th
				Birth					

[1] AI: artificial insemination.

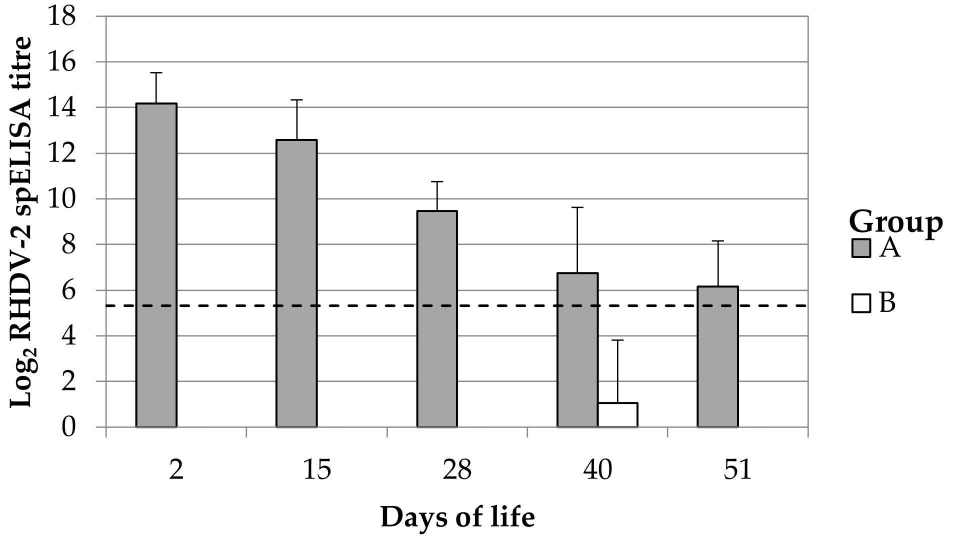

(a)

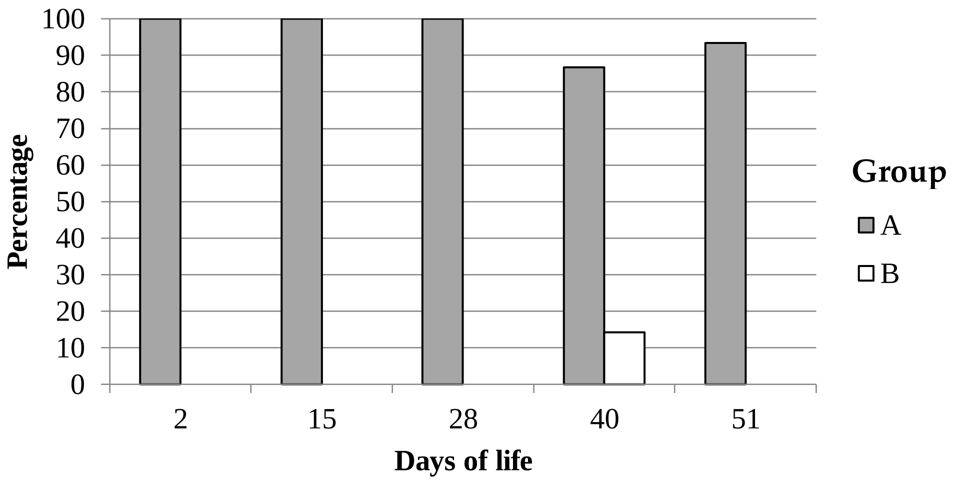

(b)

Figure S1. Duration of the maternal antibody immunity against RHDV-2 in rabbit kittens (spELISA). Results are represented as (**a**) average and standard deviation of Log_2 spELISA titres and (**b**) as percentage of positive rabbits. Rabbit kittens were born from breeding does vaccinated with either ERAVAC® (Group A) or inoculated with PBS (Group B). The slashed line indicates the cut-off of the assay (5.32 Log_2 spELISA titre).

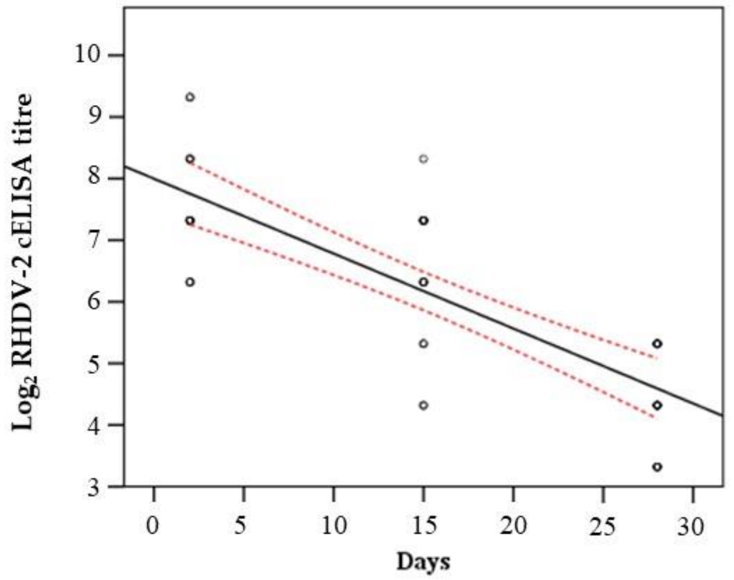

(a)

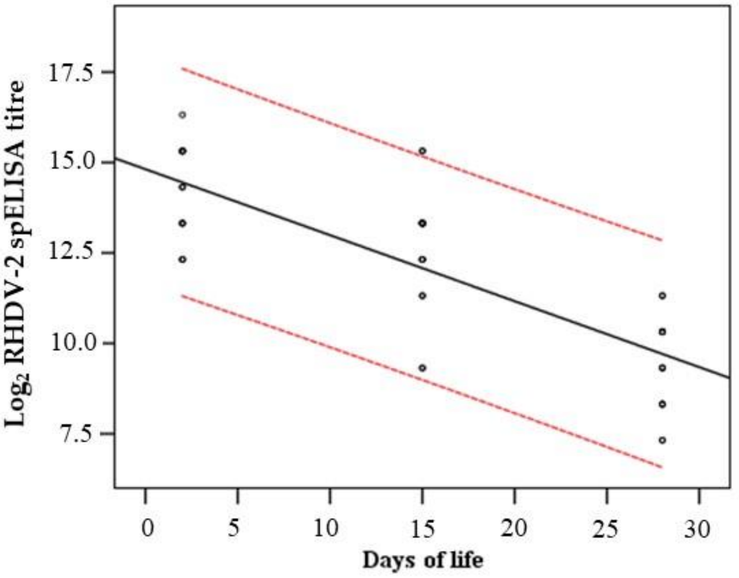

(b)

Figure S2. Dynamic of RHDV-2 antibodies in kittens; simple linear regression. Simple linear regression of the Log_2 of titres of the (**a**) cELISA (ANOVA $F(1, 42) = 68.999$, $P = 0.000$; R2 0.622) and (**b**) spELISA (ANOVA $F(1, 42) = 71.074$, $P = 0.000$; R2 0.629) values; red slashed lines represent the lower and upper limits of the 95%CI of the regression line.

Table S2. Simple linear regression coefficients.

Coefficient	Value	Standard Error	Significance	95%CI	
				Lower Limit	Upper Limit
**cELISA**					
Alpha	7.999	0.272	0.0001	7.449	8.548
Beta	0.122	0.015	0.0001	–0.151	–0.092
spELISA					
Alpha	14.814	0.402	<0.0001	14.003	15.625
Beta	–0.182	0.022	<0.00011.41×10^{-10}	–0.226	–0.139

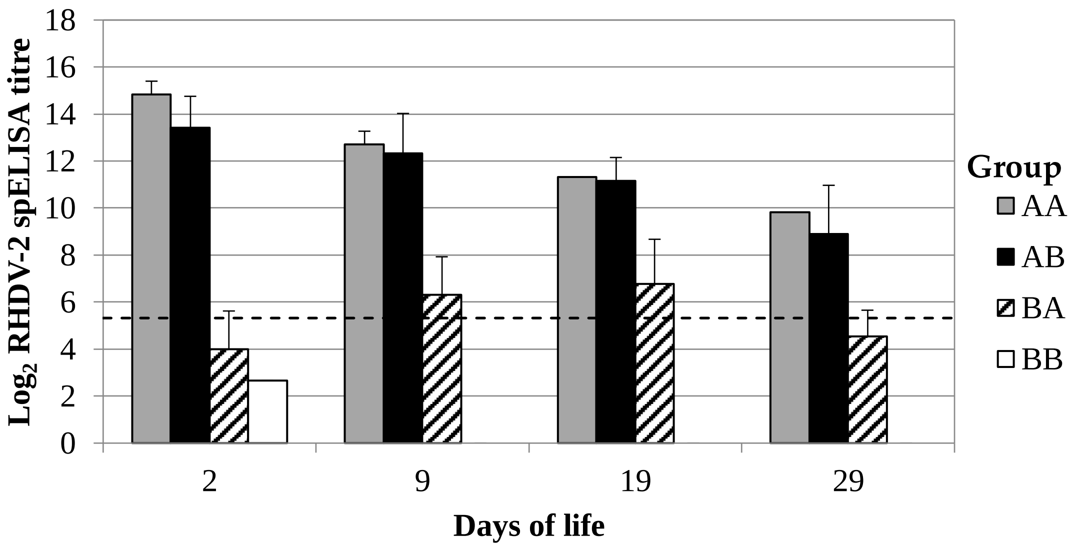

Figure S3. Mechanism of RHDV-2 antibody transmission from breeding does to rabbit kittens (spELISA). Results are represented as average and standard deviation of Log_2 spELISA titres. The rabbit kittens were cross fostered between ERAVAC® (**AA**) vaccinated, PBS inoculated breeding does (**BB**) or between them (**AB, BA**). The slashed line indicates the cut-off of the assay (5.32 Log_2 spELISA titre). In group BB just few samples were tested (n = 2) and at 9 days of life no sample was tested.
